# Supplementary material for: Role and mechanism of NCAPD3 in promoting malignant behaviors in gastric cancer
Source: Front Pharmacol. 2024 Apr 22;15:1341039. doi: 10.3389/fphar.2024.1341039 (PMC11070777; doi:10.3389/fphar.2024.1341039)
Supplement: Supplementary file 11 [file DataSheet2.ZIP › GSEA/Oncogenic signature/my_analysis.Gsea.1599461962066/PRC1_BMI_UP.V1_UP.html]

Details for gene set PRC1\_BMI\_UP.V1\_UP[GSEA]

|  || Dataset | filtered\_dataset.sample\_info.cls#WT\_versus\_NCAPD3\_MUT |
| Phenotype | sample\_info.cls#WT\_versus\_NCAPD3\_MUT |
| Upregulated in class | WT |
| GeneSet | PRC1\_BMI\_UP.V1\_UP |
| Enrichment Score (ES) | 0.2049491 |
| Normalized Enrichment Score (NES) | 0.8025283 |
| Nominal p-value | 0.7059829 |
| FDR q-value | 0.9267183 |
| FWER p-Value | 1.0 |
Table: GSEA Results Summary

  

Fig 1: Enrichment plot: PRC1\_BMI\_UP.V1\_UP      
 Profile of the Running ES Score & Positions of GeneSet Members on the Rank Ordered List

  

| SYMBOL | TITLE | RANK IN GENE LIST | RANK METRIC SCORE | RUNNING ES | CORE ENRICHMENT || 1 | 160518 | DENND5B | 13 | 1.060 | 0.0982 | Yes |
| 2 | 51351 | ZNF117 | 14 | 1.052 | 0.2049 | Yes |
| 3 | 1161 | ERCC8 | 255 | 0.625 | 0.0963 | No |
| 4 | 1021 | CDK6 | 324 | 0.579 | 0.1063 | No |
| 5 | 1962 | EHHADH | 392 | 0.541 | 0.1132 | No |
| 6 | 2140 | EYA3 | 528 | 0.458 | 0.0629 | No |
| 7 | 6493 | SIM2 | 598 | 0.425 | 0.0565 | No |
| 8 | 9949 | AMMECR1 | 650 | 0.400 | 0.0605 | No |
| 9 | 10123 | ARL4C | 957 | -0.377 | -0.1206 | No |
| 10 | 29948 | OSGIN1 | 1002 | -0.406 | -0.1110 | No |
| 11 | 6286 | S100P | 1011 | -0.412 | -0.0749 | No |
| 12 | 6590 | SLPI | 1062 | -0.453 | -0.0648 | No |
| 13 | 7128 | TNFAIP3 | 1198 | -0.573 | -0.1035 | No |
| 14 | 330 | BIRC3 | 1224 | -0.600 | -0.0606 | No |
| 15 | 64854 | USP46 | 1300 | -0.702 | -0.0431 | No |
| 16 | 2591 | GALNT3 | 1403 | -1.195 | 0.0050 | No |
Table: GSEA details [plain text format]

  

Fig 2: PRC1\_BMI\_UP.V1\_UP      
 Blue-Pink O' Gram in the Space of the Analyzed GeneSet

  

Fig 3: PRC1\_BMI\_UP.V1\_UP: Random ES distribution      
 Gene set null distribution of ES for **PRC1\_BMI\_UP.V1\_UP**
